# Lymphedema in three previously *Wuchereria bancrofti*-endemic health districts in Mali after cessation of mass drug administration

**DOI:** 10.1186/s12879-020-4777-6

**Published:** 2020-01-15

**Authors:** Housseini Dolo, Yaya Ibrahim Coulibaly, Fatoumata Nene Konipo, Siaka Yamoussa Coulibaly, Salif Seriba Doumbia, Moussa Brema Sangare, Lamine Soumaoro, Michel Emmanuel Coulibaly, Abdallah Amadou Diallo, Yaye Diarra, Modibo Sangare, Seydou Doumbia, Robert Colebunders, Thomas B. Nutman

**Affiliations:** 1 Filariasis Unit, International Center of Excellence in Research, Faculty of Medicine and Odontostomatology, BP :1805, Point G, Bamako, Mali; 2 Global Health Institute, Faculty of Medicine & Health Sciences, University of Antwerp, Antwerp, Belgium; 3 Centre National d’Appui à la lutte contre la Maladie, Bamako, Mali; 4 Laboratory of Parasitic Diseases, National Institute of Allergy and Infectious Diseases, National Institutes of Health, Bethesda, MD 20892 USA

**Keywords:** Lymphedema, Distribution, Clinical investigation, Mali, Active and passive case detection

## Abstract

**Background:**

Lymphedema is a public health problem in countries with lymphatic filariasis (LF) including Mali. We studied the epidemiology and clinical presentation of lymphedema in three previously LF-endemic health districts of Mali after at least five consecutive rounds of mass drug administration (MDA) with albendazole and ivermectin.

**Methods:**

From 2016 to 2018, we used passive and active case finding methods to identify lymphedema cases in three health districts with high pre-MDA LF prevalence: Kolondieba (66%), Bougouni (44%) and Kolokani (34%).

**Results:**

Three hundred and thirty nine cases of lymphedema were identified, 235 (69.32%) through active case finding. Their median age was 56 years (range 2–90) and 286 (84.36%) were women. Lymphedema was reported in 226 (78.5%) people aged 41 years and older compared to 73 (21.5%) people below the age of 41 years (Chi^2^ = 17.28, df = 5, *p* = 0.004). One hundred and seventy five cases of lymphedema were found in Kolondieba (66 per 100,000 people), 116 in Bougouni (19 per 100,000) and 48 in Kolokani (16 per 100,000). Stage III lymphedema was observed in 131 (38.64%), stage II in 108 (31.86%), stage IV in 46 (13.57%), stage I in 23 (6.78%), stage V in 21 (6.19%) and stage VI in ten (2.95%). In the three study districts, lymphedema affected the legs in 281 (82.89%), the arms in 42 (12.39%) and both in 16 (4.72%) (Chi2 = 13.63, *p* = 0.008).

**Conclusion:**

Health districts in Mali with the highest pre-MDA LF prevalences had the highest prevalence of lymphedema. Efforts to actively identify lymphedema cases should be scaled up in previous LF-endemic areas, and should be supplemented by a morbidity management and disability prevention plan at the peripheral health system level.

## Background

Lymphedema or elephantiasis results from the accumulation of interstitial fluid in the affected anatomic compartment causing localized swelling [1, 2]. Lymphatic filariasis (LF)-related lymphedema is caused by three filarial species, namely *Wuchereria bancrofti, Brugia malayi* and *Brugia timori* [3]. Podoconiosis is another condition in Africa causing lymphedema. However it occurs only in regions with both high altitude and significant rainfall and therefore, is considered not to occur in Mali [4, 5].

LF-related lymphedema affects over 15 million people worldwide [3]. In Mali, no lymphedema national surveillance system exists, but LF is considered to be implicated in most lymphedema cases. In Bamako, 0.58% of outpatients in the dermatology clinic at Gabriel Touré teaching hospital were reported to have lymphedema in 2018 [6]. As part of the country’s certification process for the elimination of lymphatic filariasis as Mali’s long-term objective, it is important to understand the burden of lymphedema and to manage this concern with the second goal of GPELF (MMDP) [3].

In LF-related lymphedema, the lymphatic system is damaged because the host either fails to modulate the inflammatory response towards either the filarial parasite or its endosymbiont *Wolbachia* or because secondary infections by bacteria and/or fungi drive the ongoing inflammatory processes [7]. Lymphedema often leads to social stigmatization, mental health problems, loss of income and increased medical expenses for patients and their caregivers [3].

LF prevalence mapping in 2004 showed that all 8 administrative regions of Mali were endemic for *W. bancrofti*, with an overall nationwide prevalence of 7.07% (1% in the north and 18.6% in the south of Mali) [8]. From 2005 to 2015, MDA with albendazole plus ivermectin decreased the prevalence of *W. bancrofti* infection to zero in Mali in adults tested for microfilaremia (Dembele, 2016 Unpublished). LF transmission assessment surveys (TAS) performed in the 3 health districts between 2010 and 2015 found antigenemia rates < 2% in 6–7 year old children suggesting the interruption of LF transmission according to World Health Organization guidelines [9].

The control of LF morbidities, particularly hydrocele and lymphedema, should be an essential component of LF elimination programme. However, in Mali, morbidity management, more particularly that of lymphedema, is not considered as a priority within a national program that focusses on the MDA strategy. Therefore the burden of disease caused by LF morbidities in Mali is unknown. We hypothesised that the highest burden of disease would be present in districts of Mali with previously high LF prevalence.

In preparation of a multi-site clinical trial number NCT02927496 registered by October 7, 2016 to investigate the impact of doxycycline on the regression of early stages of lymphedema, we screened three health districts of Mali (Kolondieba, Bougouni and Kolokani) with previously high LF prevalences, to identify lymphedema cases. In this paper, we describe the characteristics and epidemiological distribution of the lymphedema cases identified.

## Methods

### Study design and population

**Fig. 1:**
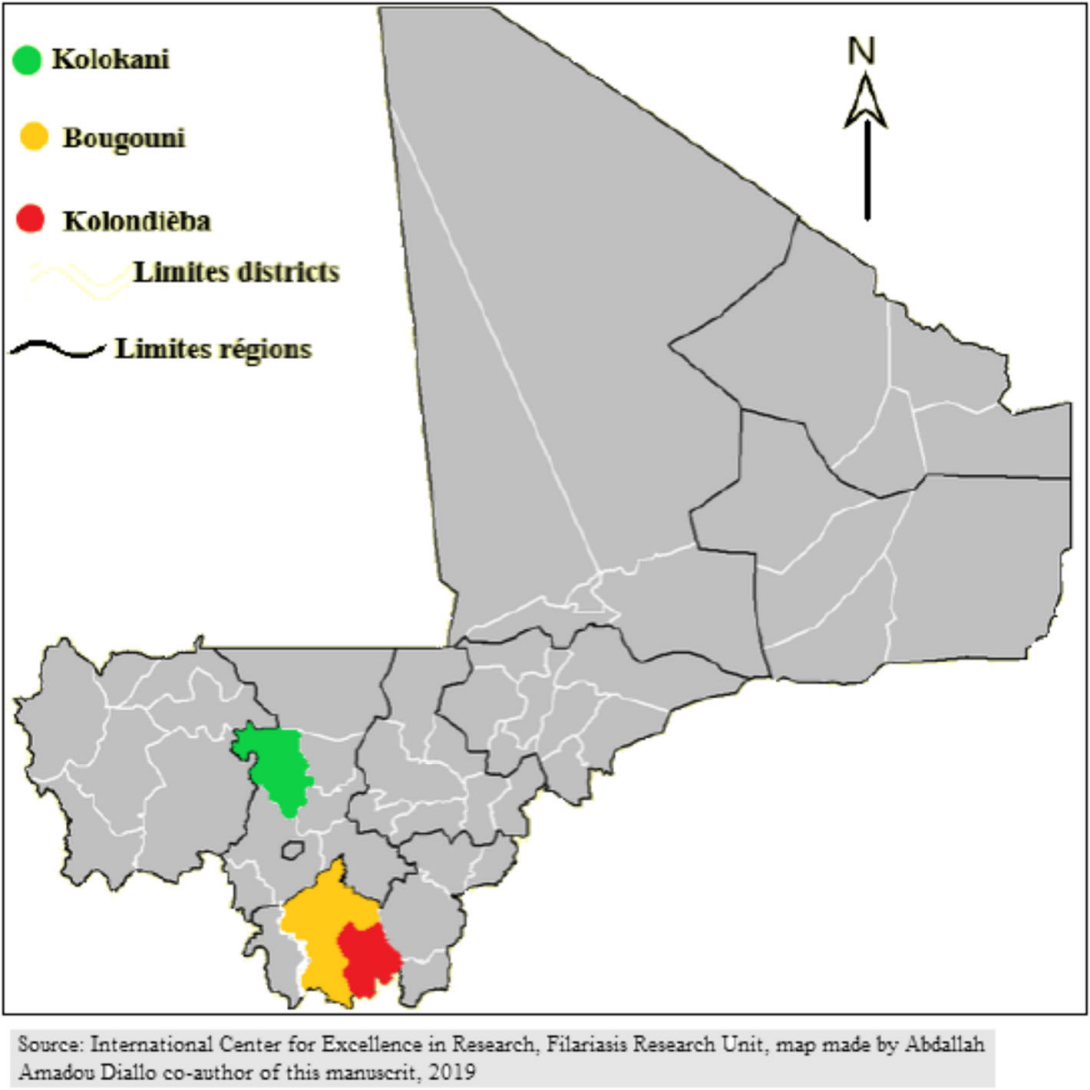
Map of Mali showing the three study districts in red (Kolondieba, Bougouni, and Kolokani) Source: International Center of Excellence in Research/Mali Filariasis research Unit, made by Abdallah Amadou Diallo co author of this manuscript, 2019

A cross sectional study was performed in the health districts of Kolondieba, Bougouni and Kolokani from August 2016 to August 2018 (Fig. 1). LF mapping in 2004 had documented a LF prevalence of 66% in Kolondieba, 48% in Bougouni and 34% in Kolokani [10]. Bougouni and Kolondieba are very large districts characterized by difficult geographic accessibility and relatively little knowledge (by the inhabitants) of the underlying causes of lymphedema.We screened for lymphedema cases using both passive and active case detection methods. With the passive case detection method, heads of community health centers and community health workers were asked to report people living with lymphedema. With the active case detection method a research team identified lymphedema cases through villages meetings, mobile phone calls and scheduled visits to remote villages.

We defined lymphedema as any non-traumatic progressive and evolving swelling of at least one upper or lower limb associated with a history of adenolymphangitis (ADL) episodes. For swelling of the lower limbs we used the Dreyer classification [11] to determine the stage of lymphedema as follows: stage I: reversible swelling that disappears spontaneously at night; stage II: non-reversible swelling that does not disappear spontaneously at night; stage III: presence of shallow skin folds; stage IV: presence of buds; stage V: presence of deep skin folds; stage VI: presence of mossy lesions; stage VII: inability to perform normal daily activities correctly and independently.

For swelling of the upper limbs we adapted the G Dreyer’s classification as follows: Stage I: swelling of an arm reported by the affected person with history of an adenolymphagitis crisis but not necessarily observed by the investigator. Stage II any swelling of an arm without visible skin folds, Stage III any swelling of an arm with at least one skin fold. When more than one member was affected we considered the most advanced stage to classify lymphedema of the person. For both legs and arms, stage I assignments were based on history.

To estimate the prevalence of lymphedema per health district we divided the number of lymphedema cases identified by all the methods (active and passive) in each health district by the 2017 population size of the district multiplied by 100,000.

### Data collection and analysis

Each case identified passively or actively was geopositioned using hand held GPS devices. We used medians to measure central tendency and chi-square test to determine statistical differences in lymphedema prevalence across categorical variables known to have a potential influence on lymphedema [12, 13], including clinical stages, different age groups and gender in all three health districts. Data were analyzed using Statistical Package for Social Sciences (SPSS) version 24.

## Results

### Characteristics of people with lymphedema

Three hundred thirty-nine people with lymphedema were identified, 175 (51.62%) in Kolondieba, 116 (34.22%) in Bougouni, and 48 (14.16%) in Kolokani (Table 2). Overall, 286 (84.36%) lymphedema cases were women (Table 1). The median age of all cases was 56 years (range 3–90). Lymphedema was reported in 266 (78.47%) people aged 41 years and above compared to 73 (21.53%) people below the age of 41 years (Chi2 = 17.28, df = 5, *p* = 0.004). All stages of lymphedema were observed except stage VII. Stage III lymphedema was observed in 131 (38.64%), stage II in 108 (31.86%), stage IV in 46 (13.57%), stage I in 23 (6.78%), stage V in 21 (6.19%) and stage VI in ten (2.95%) (Table 1).

**Table 1:** Distribution of the lymphedema cases according to gender, age and body localization in three health districts of Mali

	Kolondieba	Bougouni	Kolokani	Total	
	(*N* = 175)	(*N* = 116)	(*N* = 48)		
	Number (%)	Number (%)	Number (%)	Number (%)	*p* values
Sex					
Female	155 (88.57%)	97 (83.63%)	34 (70.83%)	286 (84.36%)	0.01
Male	20 (11.43%)	19 (16.37%)	14 (29.17%)	53 (15.64%)	
Age in years					
Median age (range)	54 (13–88)	56.5 (2–90)	56 (18–84)	56 (2–90)	0.40
Age group					
0–20	2 (1.14%)	2 (1.72%)	2 (2.08%)	5 (1.47%)	0.004
21–40	39 (22.29%)	19 (16.38%)	10 (20.83%)	68 (20.06)	
41–60	80 (45.71%)	55 (47.41%)	23 (47.92%)	158 (46.61%)	
> =61	54 (30.86%)	40 (34.48%)	14 (29.17%)	108 (31.86%)	
Anatomic location					
Legs	140 (80.00%)	104 (89.65%)	37 (77.08%)	281 (82.89%)	0.008
Arms	29 (16.57%)	8 (6.90%)	5 (10.42%)	42 (12.39%)	
Legs and Arms	6 (3.43%)	4 (3.45%)	6 (12.50%)	16 (4.72%)	
Stage					
I	13 (7.43%)	5 (4.31%)	5 (10.42%)	23 (6.78%)	0.02
II	53 (30.29%)	32 (27.59%)	23 (47.92%)	108 (31.86%)	
III	71 (40.57%)	44 (37.93%)	16 (33.33%)	131 (38.64%)	
IV	27 (15.43%)	17 (14.66%)	2 (4.17%)	46 (13.57%)	
V	8 (4.57%)	11 (9.48%)	2 (4.17%)	21 (6.19%)	
VI	3 (1.71%)	7 (6.03%)	0 (0.00%)	10 (2.95%)	

### Staging and body localization of lymphedema in the three health districts

Lymphedema affected the legs in 281(82.89%), the arms in 42 (12.39%) and both the arms and legs in 16 (4.72%) (Chi^2^ = 13.63, *p* = 0.008) (Table 1). Stage III was slightly more common in Kolondieba 71 (40.57%) and in Bougouni 44 (37.93%), stage II was most common in Kolokani 23 (47.92%) and Bougouni had the highest prevalence of stage VI 7 (6.03%). All stages were more frequent in the older age groups.

### Case detection approaches

Of the 339 people with lymphedema, only 104 (30.68%) were identified through passive case identification and 235 (69.32%) by active case identification with no statistically difference observed Chi^2^ = 3.323, df = 2, *p* = 0.18 (Table 2).

**Table 2:** Number and percentage of lymphedema cases recorded per health district and per gender according to the method of identification

Health districts	Gender	Number surveyed	Type of identification		Total
			Passive	Active	
		*n* (%)	*n* (%)	*n* (%)	
Kolondieba	Male	20 (11.42%)	7 (35.00%)	13 (65.00%)	20 (100%)
	Female	155(88.58%)	45 (29.00%)	110 (70.00%)	155 (100%)
Sub-total 1*		175 (51.62%)	52 (29.71%)	123 (70.29%)	175 (100%)
Bougouni	Male	19 (16.38%)	3 (15.79%)	16 (84.21%)	19 (100%)
	Female	97(83.62%)	29 (29.90%)	68 (70.10%)	97 (100%)
Sub-total 2*		116 (34.22%)	32 (27.59%)	84 (72.41%)	116 (100%)
Kolokani	Male	14 (29.17%)	8 (57.14%)	6 (42.86%)	14 (100%)
	Female	34(70.83%)	12 (35.29%)	22 (64.71%)	34 (100%)
Sub-total 3*		48 (14.16%)	20 (41.66%)	28 (58.34%)	48 (100%)
Total		339 (100%)	104 (30.68%)	235 (69.32%)	339 (100%)

*Subtotal proportions were on the total cases of lymphedema identified

*Per identification type difference of reported lymphedema cases in the three health district (Chi^2^ = 3.323, df = 2, *p* = 0.18)

### Estimation of lymphedema prevalence

The estimated prevalence of lymphedema in Kolondieba was 65.60 per 100,000 people, 19.17 per 100,000 people in Bougouni and 15.66 per 100,000 people in Kolokani; overall in the three health districts it was 28.77 per 100,000 people (Table 3).

**Table 3:** Estimation of lymphedema prevalence per health district in three health district of Mali

District	Population size in 2017	Prevalence of LF during the mapping in 2005^a^	Number of lymphedema carriers	Estimated lymphedema prevalence for 100,000 persons	95% CI
Kolondieba	266,753	66%	175	65.60/100,000	[55.89–75.32]
Bougouni	604,962	48%	116	19.17/100,000	[15.69–22.66]
Kolokani	306,445	34%	48	15.66/100,000	[11.23–20.09]
Total	1,178,160	49%	339	28.77/100,000	[25.71–31.84]

^a^Data are from the LF national report in 2005

### Examples of clinical presentations of lymphedema in Mali

#### Case 1

**Fig. 2:**
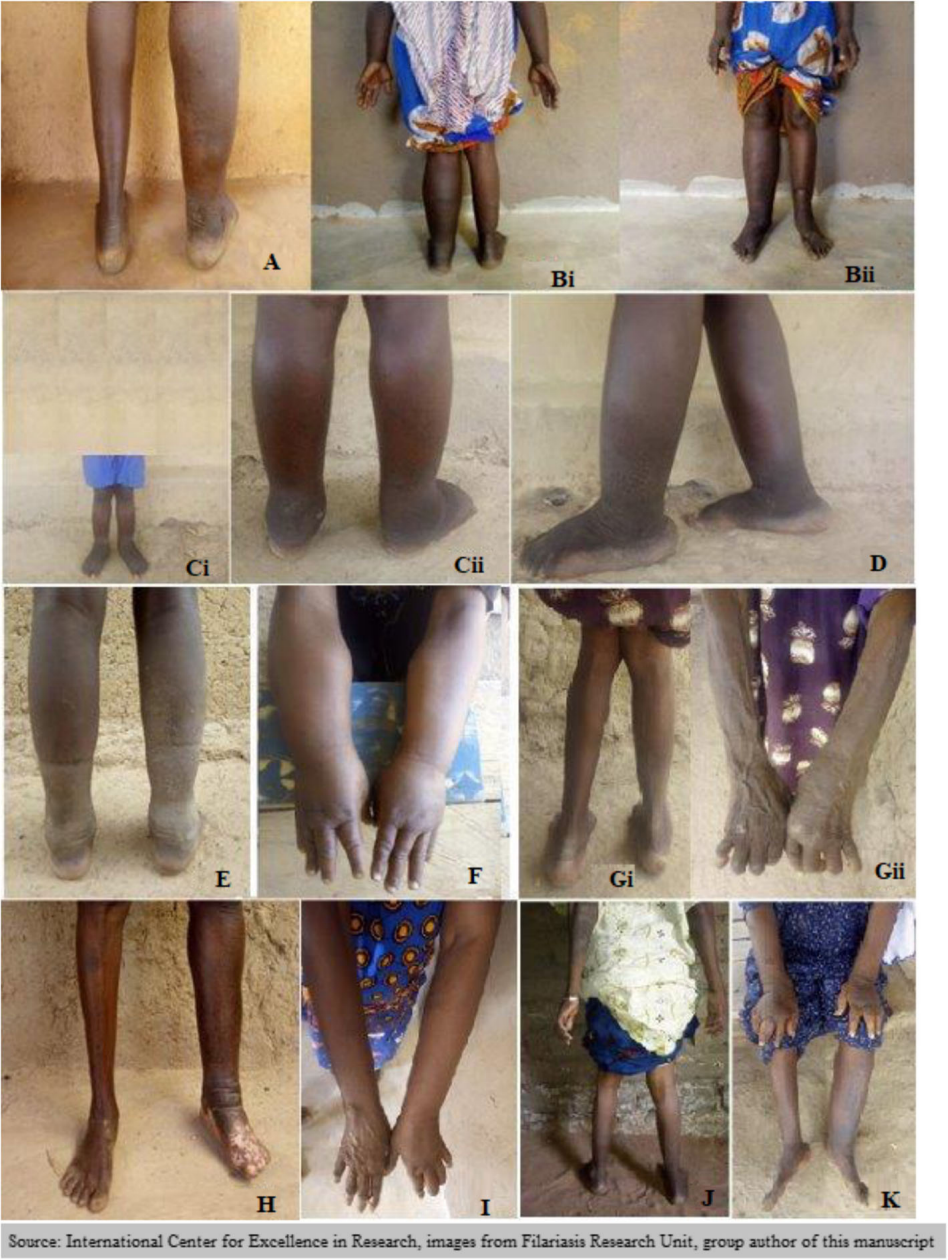
Variability of clinical presentation in patients with lymphedema in Mali. Panels showing unilateral lower extremity lymphedema (**A**), four limb lymphedema each at a different stage (**Bi**/**Bii**),familial lymphedema affected a children of less than 5 years old (**Ci**, **Cii**) and her mother (**D**), lymphedema of two legs in young man (**E**), lymphedema of two arms in woman(**F**), asymmetric lymphedema of left arm(**Gii**) and right leg (**Gi**), lymphedema of left leg with hypochromia (**H**), lymphedema of the left arm in woman (**I**), lymphedema of two legs and right arm at different stages in woman (**J**) and lymphedema of two arms and left leg in woman (**K**). Source: International Center of Excellence in Research/Mali images from Filariasis Research Unit group author of this manuscript

A woman between 30 and 40 years with stage VI lymphedema with swelling of the right leg from the foot to above the knee with mossy lesions (Fig. 2A). She reported two to three episodes of ADL per year.

#### Case 2

A woman above 65 years with lymphedema affecting both upper and lower limbs: right leg at stage III, left leg and right arm at stage II and the left arm at stage I (Fig. 2 Bi and Bii). She reported at least two episodes of ADL per year.

#### Case 3

A mother between 20 and 30 years and her less than five -year-old daughter born with bilateral and symmetrical lymphedema at the lower limbs. The mother presented with stage VI lymphedema (Fig. 2D) and the daughter with stage III lymphedema (Fig. 2 Ci and 2Cii).

#### Case 4

A man between 15 and 20 years with lymphedema affecting the two legs. The two legs were at stage V (Fig. 2E). He reported having multiple episodes of ADL.

#### Case 5

A woman above 50 years old with stage III lymphedema in two arms (Fig. 2F). She reported having multiple ADL episodes over the past 10 years.

Case 6: A woman between 60 and 70 years had lymphedema in the right leg (2Gi) and left arm (2Gii) at stage II. She had no episodes of ADL for the last 3 years and had noticed a fairly dramatic decline in lymphedema size as she aged.

#### Case 7

A woman between 60 and 70 years had a lymphedema of the left leg at stage V associated with dermal hypochromia (Fig. 2H). She reported having an average of three ADL attacks per year.

#### Case 8

A woman between 45 and 50 years with stage II lymphedema of the left arm (Fig. 2I). She had reported three ADL per year.

#### Case 9

A woman between 60 and 65 years with stage II lymphedema of the left leg and right arm and stage I lymphedema of the right leg (Fig. 2J). She had a chronic non-weeping ulceration at the medial malleolus of her left leg. She had reported having ADL attacks on average twice a month.

Case 10: A woman above 80 years old with lymphedema of two arms and one left leg at stage III (Fig. 2K). There is a history of lymphedema in her family. She had reported an average three ADL attacks per month.

## Discussion

We identified 339 cases of lymphedema, mostly through active case finding, in three previously LF hyper-endemic health districts (Kolondieba, Bougouni, and Kolokani) in Mali. Most people with lymphedema were identified in Kolondieba, the district previously reported with the highest pre-MDA LF prevalence (66%) [10]. Lymphedema was found predominantly among those in the older age group (median age 56 years); most cases occured in women (84%). The preponderance of lymphedema among women is in line with observations in other LF endemic countries [12, 14, 15]. The age and gender distribution is different from what has been reported in podoconiosis which is mainly observed in individuals between 10 and 30 years old without any gender predominance [4, 13, 16].

Lymphedemas were more frequently observed in those older than 41 years than in those in the lower age groups. We only observed 1 case of lymphedema (in the health district of Bougouni) in the age group of below 6 years after the MDA stopped in 2015. This difference is most likely a consequence of ivermectin- and albendazole-based MDA whereby the younger age groups had reduced exposure to LF parasites and therefore being less suspectible to develop lymphedema [17].

Most late stages of lymphedema were identified in Bougouni and Kolondieba, very large districts characterized by difficult geographic accessibility; these remote areas are locations in which lymphedema is believed to be caused by evil or malediction [18]. It is against this backdrop that our study population may have been difficult to mobilize, which in turn may have underestimated the true burden of lymphedema.

Our study highlights the importance of conducting active case detection which was more successful (approximatively 70% of cases detected) than passive case detection. This is explained by the fact that people with lymphedema usually consider their condition to be incurable and therefore may not consult health workers. Moreover, stigma concerning lymphedema may also play a role in further reducing uptake of community health services [19, 20].

We observed several uncommon cases of lymphedema such as a case of congenital lymphedema also known as Milroy disease (case 3), an autosomal dominant disorder that causes lymphatic vessel dysfunction or absence of functional lymphatics [21]. We also observed a case of lymphedema affecting both upper and lower limbs at different stages (case 2) and a case of lymphedema affecting symmetrically two legs in a 18 year old young man without multiple hard skin nodules like podoconiosis (case 4) and those cases seem not related to LF [22].

In tropical countries, LF and podoconiosis are the most frequent causes of acquired lymphedema as opposed to European countries and in the United States where cancer-related treatment is the most frequent cause [23]. Medical record review of 511 patients with lymphedema attending a dermatological clinic in Tigray, Ethiopia between 2005 and 9, revealed that 9.2% of them were people with LF related lymphedema [24]. Intergrated morbidity mapping of LF and podoconiosis in 20 co-endemic districts in Ethiopia in 2018 detected 26,123 cases of lymphedema, 89.3% with bilateral lymphedema [25]. The prevalence of men reporting hydrocoele was low, 2.4 per 10,000 population. This large number of bilateral lymphedema cases and the low prevalence of hydrocoele suggest that most lymphedema cases in Ethiopia are caused by podoconiosis.

In Mali, LF control programs, akin to elsewhere in Africa, are more focused on MDA to interrupt transmission than on lymphedema morbidity management [26]. In a study conducted in Togo in 2007, only 28.2% of 188 lymphedema cases were reported to have benefited from some sort of treatment. As lymphedema is a chronic, progressive condition, it is important to implement a morbidity management and disability prevention (MMDP) programme for LF as recommended by the WHO [26].

As limitations of this study, we need to mention that we did not perform a door-to-door survey and therefore we may have underestimated the prevalence of lymphedema. We also focused on case finding and did not assess co-morbidities such as scrotum swelling-hydrocele, treatment practices nor assessed the perception of communities about lymphedema.

## Conclusion

Lymphedema remains a public health problem in previously LF endemic regions in Mali where transmission has been interrupted. Consequently it is imperative that active lymphedema case identification be scaled up in all previously LF endemic regions in Mali and that a MMDP programme at peripheral health system level is implemented to meet LF elimination goal in near future.

## Data Availability

The datasets related to this paper are available through the NIAID/Mali International Center of Excellence in Research.

## Abbreviations

ADL: Adenolymphangitis
FMOS: Faculté de Médicine et d’Odontostomatologie (Faculty of Medicine and Odontostomatology)
LF: Lymphatic filariasis
MDA: Mass drug administration
MMDP: Morbidity management and disability prevention
TAS: Transmission assessment surveys
